# Spatio‐temporal trends in caries: A study on children in Berlin‐Mitte

**DOI:** 10.1002/cre2.354

**Published:** 2020-11-17

**Authors:** Ae Kyung Lee, Annette Aigner, Timo Schmid, Tobias Kurth

**Affiliations:** ^1^ Institute of Public Health Charité ‐ Universitätsmedizin Berlin Berlin Germany; ^2^ Institute of Biometry and Clinical Epidemiology Charité ‐ Universitätsmedizin Berlin Berlin Germany; ^3^ Berlin Institute of Health (BIH) Berlin Germany; ^4^ Institute of Statistics and Econometrics Freie Universität Berlin Berlin Germany

**Keywords:** children, dental caries, health disparities, routinely collected data, small‐area analysis, spatio‐temporal analysis

## Abstract

**Background:**

Significant inequalities in caries distribution among children in Germany have been reported, but small‐scale areas remain understudied.

**Aim:**

To examine spatio‐temporal trends in children's dental caries at the small‐area level in Berlin‐Mitte.

**Design:**

Routinely collected data from Berlin's annual Health Examination Surveys were used, which also include information on age, sex, country of origin, and residential area. The study population consists of 14,866 children aged 5 to 7 between 2006 and 2014 in the district of Berlin‐Mitte. Outcome variables are the dmft (decayed, missing, and filled teeth), the presence of any caries experience, untreated caries, and caries risk. The outcomes are summarized descriptively and graphically presented for 10 quarters and 41 communities within Berlin‐Mitte.

**Results:**

Relevant gaps in children's dental caries were discovered between the quarters of Mitte. Three quarters in the northeast part of Mitte have consistently indicated the lowest oral health status in all four outcomes, and children having high caries risk have been increasingly concentrating in this area over time. Despite the continuous improvements in the southern part, the averages in total of Mitte for all outcomes have risen.

**Conclusion:**

Our findings confirm the spatiotemporally mounting disparities in children's oral health between the quarters in Berlin‐Mitte and that particular quarters need urgent attention. The small‐area approach made it easier and more effective to reveal the spatial distribution of children's dental caries at the local level. The small‐area analysis should be strongly encouraged in future caries research to narrow the inequalities in children's oral health.

## 1 INTRODUCTION

Germany, along with other high‐income countries, has shown continuous improvements in children's oral health during the past decades (Jordan & Micheelis, 2016; OECD, 2009). However, several studies observe that a steady downward trend in caries experience for the primary dentition in Europe including Germany stopped in the early 2000s, while the descending trend for the permanent dentition was continuous (Haugejorden & Birkeland, 2002; Splieth et al., 2017). Other studies report that oral health among children generally improved, but its inequalities are widening (Kramer, Hakeberg, Petzold, & Ostberg, 2016; Sengupta, Christensen, Mortensen, Skovgaard, & Andersen, 2017). The polarization has been growing in Germany as well, and particularly in Berlin, the capital of Germany, there is a large gap in oral health between children belonging to different social strata (Pieper, 2005; Splieth et al., 2017).

Berlin exhibits a significantly lower level of children's oral health than other regions in Germany (Splieth et al., 2017). Annual reports of Berlin point out that a great difference in children's oral health among districts of Berlin exists (Bettge & Oberwöhrmann, 2015). The district Mitte in the center of Berlin is especially prominent in this respect, as the general health status, as well as oral health have been found to be much poorer compared to other districts. Mitte is also characterized by a very high proportion of families who are immigrants and who are socially underprivileged (Butler, Uhlig, & Brockstedt, 2009).

**FIGURE 1:**
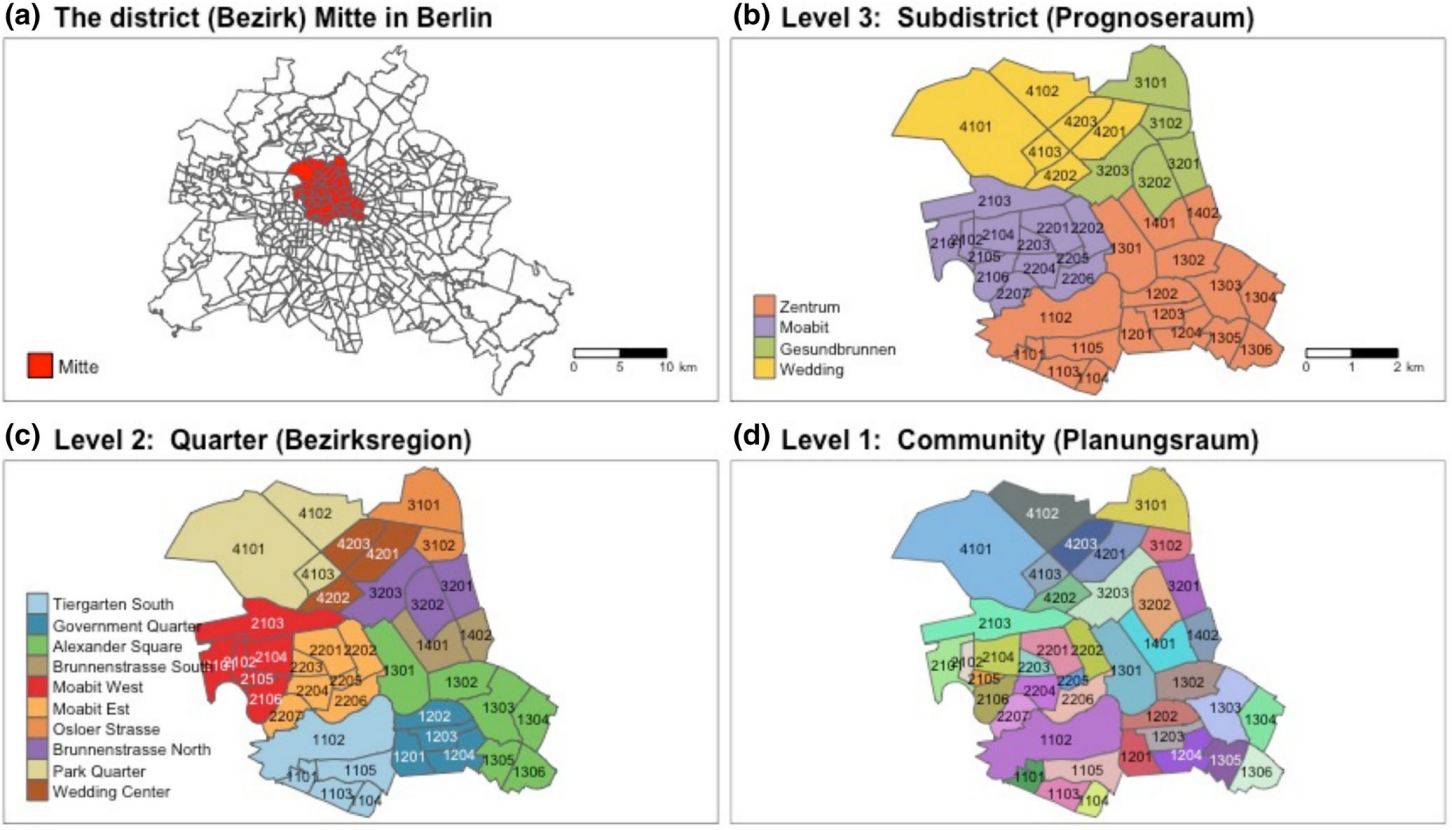
Berlin’s administrative boundary: (a) The district (*Bezirk*) Mitte in Berlin, (b) Level 3: Subdistrict (*Prognoseraum*), (c) Level 2: Quarter (*Bezirksregion*), (d) Level 1: Community (*Planungsraum*)

Berlin has its own administrative system. Each of the 12 districts is divided into several subdistricts (Prognoseräume), which are then formed by quarters (Bezirksregionen). The smallest administrative unit are communities (Planungsräume). Mitte consists of four subdistricts (Zentrum, Moabit, Gesundbrunnen, and Wedding), 10 quarters, and 41 communities (Figure 1). The majority of dental studies, including studies from Germany (Jordan & Micheelis, 2016; Splieth et al., 2017), have been based on a large‐area approach by focusing on a country, a region, or a city. Most existing health reports from Berlin also conducted their analyses at the levels of districts, or occasionally of subdistricts (Bettge & Oberwöhrmann, 2015; Lakes & Burkart, 2016).

Recently, the small‐area approach is being used frequently in other disciplines (Lothrop, Hussaini, Billheimer, & Beamer, 2017), but it is rarely applied in dental research. The small‐area analysis reveals specific problems in specific areas, and as such identifies high‐risk areas more accurately than research at national or regional levels, helping to ensure that appropriate interventions are implemented where they are most needed (Piel et al., 2020). Identifying spatial disparities in oral health within small‐scale areas can be an important basis to find hidden risk factors contributing to oral health inequalities. Therefore, the purpose of this exploratory study is to examine spatio‐temporal trends in dental caries among first‐year schoolchildren at the level of small areas in Berlin‐Mitte. We investigate the spatial distribution of dental caries within Berlin‐Mitte, the temporal patterns from 2006 to 2014, as well as the spatio‐temporal dynamics.

## 2 MATERIALS AND METHODS

The district Mitte accounts for 4.4% (39.47 km^2^) of the total area of Berlin and had about 380,000 inhabitants, while Berlin had a total population of 3.7 million in 2018 (Amt für Statistik Berlin‐Brandenburg, 2018). Mitte is the second smallest of the 12 districts in Berlin, but the most preferred among foreigners without German citizenship such that 17.6% of the foreigners living in Berlin settled down in Mitte, and the percentage of Mitte's foreign residents is increasing annually. In 2018, Mitte was the district with the highest proportion (33.6%) of foreign inhabitants in Berlin and had the second‐highest population density (Amt für Statistik Berlin‐Brandenburg, 2018).

The anonymized data for our secondary analysis was derived from routinely collected data, which were generated by the annual health examination mandatory for children entering primary school in Berlin. Lately, routinely collected data (RCD) have been used more often in the biomedical field due to their advantages such as representativeness and generalizability, coverage of large populations, low cost, and longitudinal structure (Benchimol et al., 2015). But there are also several limitations in RCD, such as uncertain validity and lacking information on key confounders or risk factors due to the finite number of assessed characteristics (Jorm, 2015). Our data was gathered through a dental examination and a questionnaire answered by parents. Children were clinically examined by qualified dentists working at the Health Center of Mitte. This cross‐sectional survey is repeated annually with a target population consisting of children between 5 and 7 years living in Mitte. We analyzed the data of Berlin‐Mitte from 2006 to 2014. Furthermore, children who did not provide residency information were excluded from the analysis. The data comprised 10 quarters and 41 communities (Figure 1).

In the original survey a variety of variables was collected. Of importance for this research were demographic variables such as age, sex, and country of origin. The latter was classified into several groups: Germany, Arab countries, Turkey, Eastern European countries, Western European countries, other countries, and we additionally investigated observations with missing information on their country of origin. Since 2010, non‐response to the question on the country of origin has not occurred due to systematic changes in the survey process. However, we included this group in our exploratory study, as it could provide important information for further detailed analysis. The assignment of origin was carried out by examiners according to the criteria proposed by the Berlin Senate Administration for Health and Social Affairs (Bettge & Oberwöhrmann, 2015). The anonymized dataset containing no personal information was obtained from the Bezirksamt Berlin‐Mitte in accordance with the Raw‐Material‐Use Regulations. As this research uses anonymized data, ethical approval was not necessary. This study is described following the STROBE checklist recommendations.

The study investigated four outcome variables measuring dental caries. The first outcome is the dmft (decayed, missing, and filled teeth in primary dentition) index as a discrete variable, which was suggested by the World Health Organization (Petersen, Baez, & WHO, 2013). The second outcome is the presence of any caries experience as a binary variable (yes/no). It was used to judge the existence of life‐long caries experience in primary or permanent teeth by whether the sum of dmft and DMFT (decayed, missing, and filled teeth in permanent dentition) was greater than or equal to zero. Children whose dmft and DMFT scores are all zero are called caries‐free. Germany is aiming at raising the proportion of caries‐free children among 6‐year‐olds up to 80% by 2020 (Ziller, Micheelis, Oesterreich, & Reich, 2006). Based on this cut‐off, areas in which 20% or fewer children have any caries experience are considered as being in a good oral health condition. The third outcome is untreated caries as a discrete variable, which is defined as the sum of dt and DT (decayed teeth in permanent dentition) components of dmft/DMFT index. It indicates the total number of untreated decayed teeth at the time of examination. Caries risk as a binary variable (yes/no) represents the fourth outcome, measured by the criteria of the German Working Group for Youth Dental Care (DAJ, 2000). According to this criteria, 5‐year‐olds with dmft >4 and 6‐7‐year‐olds with dmft/DMFT >5 or DT > 0 were identified as being at high risk of caries. For children belonging to this group in Germany, the participation in the intensive prophylaxis program is strongly recommended. Therefore, the outcome of caries risk can be used to identify children needing substantial treatment.

Characteristics of children are summarized with descriptive statistical methods. We report frequencies and percentages, as well as the mean and standard deviation (SD) for the outcomes and explanatory variables of this research and visualize them through maps and graphs to see the spatial, temporal, and spatio‐temporal trends. All analyses and visualizations were performed using R (version 3.5.2) (R Core Team, 2018) and the R packages *tmap* (v.2.2) (Tennekes, 2018), and *tidyverse* (v.1.2.1) (Wickham et al., 2019).

## 3 RESULTS

Our study included a total of 14,866 children examined between 2006 and 2014, with an annual average of 1,652 (Table 1 and Appendix Table). Out of 14,933 original participants, 67 had to be excluded from the analysis as they were not living in Berlin‐Mitte or did not give any information about their living quarter and community. The mean age of participants was 5.6 years, the number of 7‐year‐olds was less than 1% of all children. The sex ratio was relatively well‐balanced, there were slightly more boys than girls every year except for 2010, with an average of 50.9% for boys and 49.1% for girls. In terms of the children's country of origin, the proportion of Turkish origin was the highest (31.8%), children of German origin were about a quarter (25.6%), followed by Arab origin (15.1%).

**TABLE 1:** Oral health status of study population in Berlin‐Mitte, 2006–2014

	Total	dmft	Any caries experience^a^	Untreated caries^b^	Caries risk^c^
	n (%)	Mean (SD)	n (%)	Mean (SD)	n (%)
All participants	14,866	2.87 (3.30)	9,360 (63.0)	1.33 (2.15)	3,748 (25.2)
Sex					
Male	7,574 (50.9)	3.07 (3.44)	4,884 (64.5)	1.41 (2.23)	2,070 (27.3)
Female	7,292 (49.1)	2.65 (3.13)	4,476 (61.4)	1.24 (2.06)	1,678 (23.0)
Age					
5	6,424 (43.2)	2.77 (3.32)	3,930 (61.2)	1.21 (2.04)	1,687 (26.3)
6	8,307 (55.9)	2.93 (3.28)	5,334 (64.2)	1.41 (2.22)	2,027 (24.4)
7	135 (0.9)	3.43 (3.59)	96 (71.1)	1.95 (2.65)	34 (25.2)
Origin					
German	3,802 (25.6)	1.93 (2.87)	1,854 (48.8)	0.78 (1.61)	603 (15.9)
Turkish	4,720 (31.8)	3.06 (3.30)	3,179 (67.4)	1.43 (2.14)	1,281 (27.1)
Arab	2,238 (15.1)	3.24 (3.30)	1,570 (70.2)	1.47 (2.20)	676 (30.2)
Eastern European countries	1,849 (12.4)	4.12 (3.60)	1,421 (76.9)	2.21 (2.80)	703 (38.0)
Western European countries	616 (4.1)	2.48 (3.28)	347 (56.3)	1.11 (1.97)	132 (21.4)
Other countries	1,563 (10.5)	2.65 (3.24)	937 (59.9)	1.16 (1.98)	328 (21.0)
Not answered	78 (0.5)	3.58 (3.58)	52 (66.7)	2.18 (2.87)	25 (32.1)
School year					
2006	1,719 (11.6)	2.99 (3.42)	1,094 (63.6)	1.65 (2.50)	450 (26.2)
2007	1,144 (7.7)	3.11 (3.36)	771 (67.4)	1.65 (2.33)	322 (28.1)
2008	1,714 (11.5)	2.94 (3.23)	1,130 (65.9)	1.30 (2.13)	447 (26.1)
2009	1,927 (13.0)	2.74 (3.27)	1,168 (60.6)	1.24 (2.12)	465 (24.1)
2010	1,989 (13.4)	2.55 (3.12)	1,188 (59.7)	1.15 (1.94)	421 (21.2)
2011	1,587 (10.7)	2.76 (3.22)	990 (62.4)	1.15 (2.02)	383 (24.1)
2012	2,016 (13.6)	2.79 (3.26)	1,218 (60.4)	1.19 (2.03)	496 (24.6)
2013	1,658 (11.2)	2.91 (3.39)	1,046 (63.1)	1.30 (2.06)	430 (25.9)
2014	1,112 (7.5)	3.30 (3.49)	755 (67.9)	1.55 (2.23)	334 (30.0)
Quarter					
Tiergarten South	312 (2.1)	2.72 (3.06)	208 (66.7)	1.30 (2.05)	75 (24.0)
Government Quarter	202 (1.4)	2.10 (2.89)	102 (50.5)	0.84 (1.63)	37 (18.3)
Alexander Square	809 (5.4)	1.96 (2.79)	410 (50.7)	0.75 (1.51)	119 (14.7)
Brunnenstrasse South	734 (4.9)	1.47 (2.48)	310 (42.2)	0.54 (1.43)	78 (10.6)
Moabit West	1,764 (11.9)	2.46 (3.25)	955 (54.1)	1.14 (2.08)	370 (21.0)
Moabit Est	1,683 (11.3)	2.15 (2.93)	893 (53.1)	0.95 (1.81)	290 (17.2)
Osloer Strasse	2,247 (15.1)	3.73 (3.48)	1,674 (74.5)	1.93 (2.48)	797 (35.5)
Brunnenstrasse North	2,468 (16.6)	3.36 (3.45)	1,724 (69.9)	1.52 (2.25)	752 (30.5)
Park Quarter	1,616 (10.9)	2.63 (3.26)	961 (59.5)	1.08 (1.97)	368 (22.8)
Wedding Center	3,031 (20.4)	3.22 (3.33)	2,123 (70.0)	1.55 (2.26)	862 (28.4)

Abbreviations: dmft, decayed, missing, and filled teeth; SD, standard deviation.

^a^ Any caries experience is defined as the presence of any caries experience in primary or permanent teeth (yes/no).

^b^ Untreated caries is defined as the total number of untreated decayed teeth in primary or permanent dentition at the time of examination.

^c^ Caries risk is defined as the presence of high caries risk (yes/no) according to the criteria of DAJ (DAJ, 2000).

The total mean dmft was 2.87, the average proportion of children with any caries experience was 63% (Table 1). The mean number of untreated decayed teeth was 1.33, the average proportion of children with caries risk was 25.2%. Boys reported a higher dmft than girls (mean: 3.07 vs 2.65) (Table 1). In tendency, the older the children, the higher the dmft, as it was 3.43 among 7‐year‐olds in contrast to values of 2.77 among 5‐year‐olds and 2.93 among 6‐year‐olds.

**FIGURE 2:**
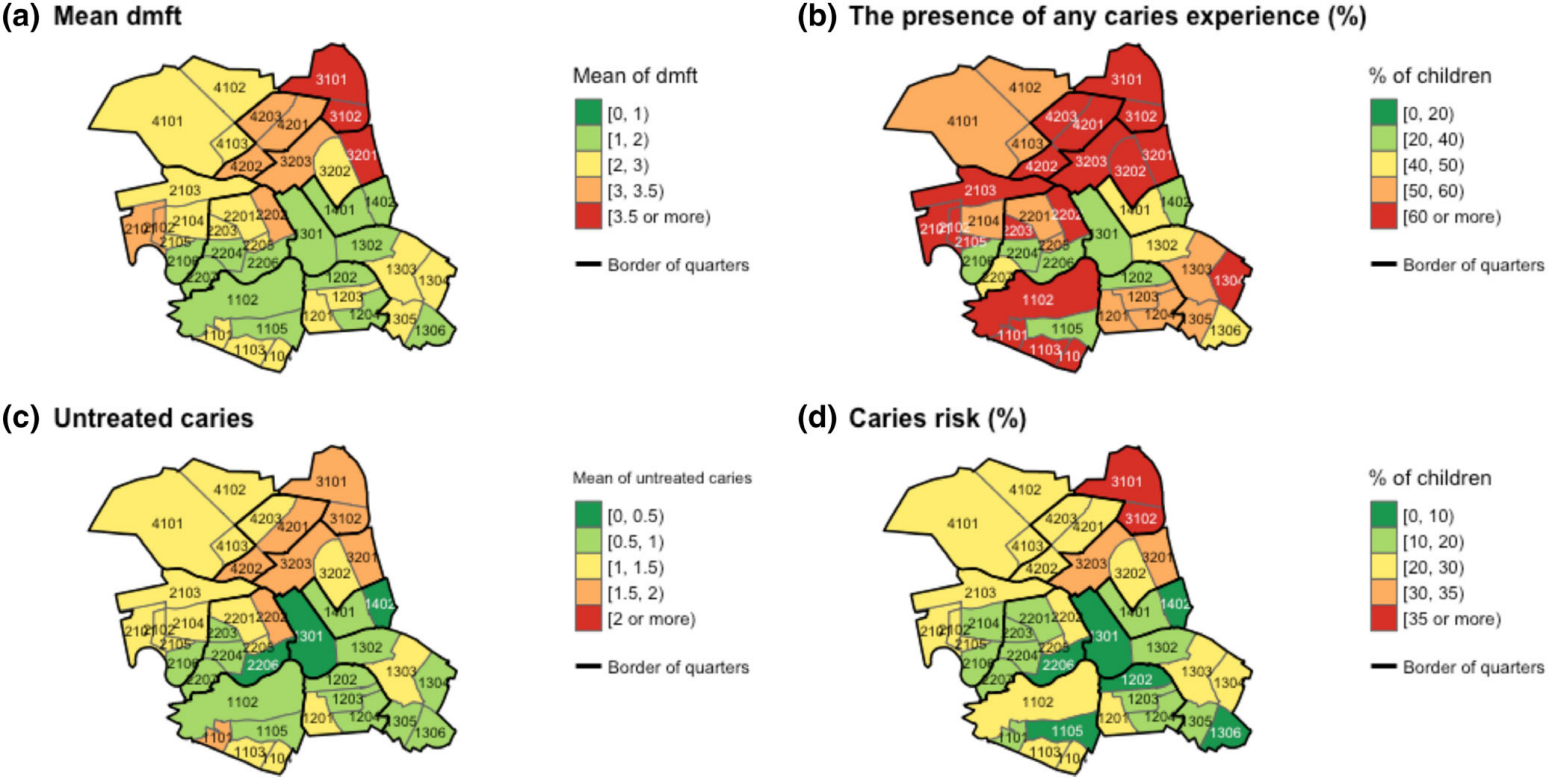
Spatial distribution of the four outcomes at community level in Berlin‐Mitte during 2006–2014: (a) Mean dmft (decayed, missing, and filled teeth), (b) The presence of any caries experience, (c) Untreated caries, and (d) Caries risk. Black boundaries indicate the ten quarters of Mitte; Gray boundaries indicate the forty‐one communities of Mitte. The first two digits of four‐figure codes meaning each community on these maps indicate its quarter

The spatial distribution of the four outcomes during the entire period is shown in Figure 2. The maps depict the mean dmft, the average presence of any caries experience, the average untreated caries, and the average caries risk among children by community. The whole subdistrict of Gesundbrunnen (Quarters 31 and 32) located in the northeast of Mitte and its neighbouring Wedding Center (Quarter 42) presented the lowest level of oral health. Among them, Osloer Strasse (Quarter 31) showed an average dmft of 3.73 (SD 3.48), an average presence of any caries experience of 74.5%, an average untreated caries of 1.93 (SD 2.48), and an average caries risk of 35.5%. These values were the worst in the whole district of Mitte for each indicator. Wedding Center and Brunnenstrasse North (Quarter 32), both of which are bordered by Osloer Strasse, featured a quite low level of oral health overall. The oral health status of Northern Moabit and Southern Zentrum was also relatively low. On the contrary, a horizontal zone is observed in the center of Mitte, which is most clearly seen in Figure 2b, with a very good level in all four indices. Nevertheless, there was no quarter where caries‐free children amounted to 80% or above. The highest value was 57.8% in Brunnenstrasse South (Quarter 14).

**FIGURE 3:**
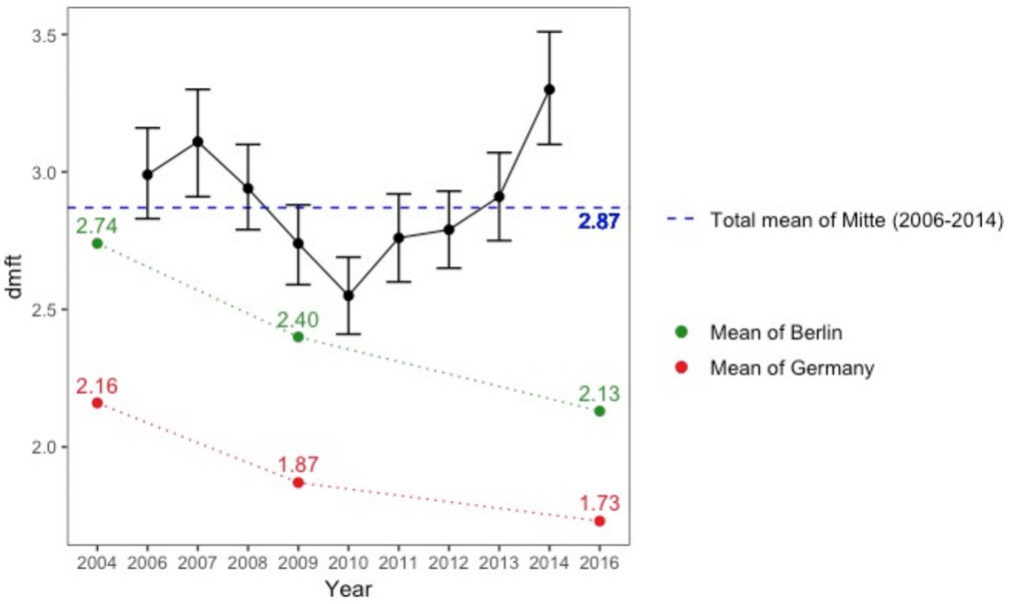
Temporal pattern of mean dmft (decayed, missing, and filled teeth) with 95% confidence interval in Berlin‐Mitte from 2006 to 2014. Total means of Berlin and Germany examined in 2004, 2009, and 2016 are plotted alongside

The four outcomes displayed a common pattern for temporal change (Table 1). The mean dmft of Mitte gradually decreased from 2007 to 2010, but increased since then until 2014 (Figure 3). Particularly, the values of 2014 compared to 2013 increased by 0.4 units (13%) in dmft, by 0.25 units (19%) in the number of untreated decayed teeth, and by more than 4% in the presence of any caries experience and high caries risk. In this regard, it is notable that the steady decline in dmft of Germany and Berlin and the rapidly deteriorating status of Mitte are in stark contrast.

**FIGURE 4:**
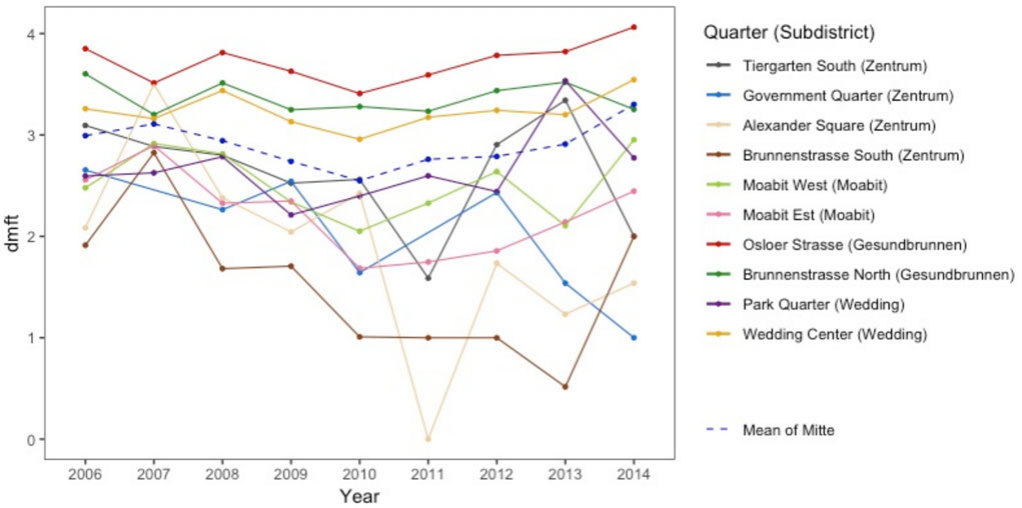
Trends in mean dmft (decayed, missing, and filled teeth) for each quarter in Berlin‐Mitte from 2006 to 2014

The spatio‐temporal variation in dmft among the 10 quarters of Mitte is plotted in Figure 4 and Appendix Figure 1. Osloer Strasse of Gesundbrunnen consistently presented the highest dmft, which was even rising. On the other hand, the dmft scores of most quarters in the subdistricts such as Zentrum and Moabit showed a gradually declining trend, except for 2014, which features the sharpest deterioration for the whole of Mitte. The total mean during nine years in Osloer Strasse was 3.73, contrary to 1.47 in Brunnenstrasse South. In 2014, the difference widened further such that dmft of Osloer Strasse reached 4.06, in contrast to 1.00 of Government Quarter. In other words, there were great differences in mean dmft between quarters and the discrepancies were becoming even greater over time. The dmft of Tiergarten South and Government Quarter, which had fewer participants, were fluctuating.

A similar tendency is found in the presence of any caries experience (Appendix Figure 2) and untreated caries (Appendix Figure 3). The average presence of any caries experience was greater than 50% in every quarter of Mitte until 2008, since then the averages of several quarters have been reduced. However, the averages of three quarters, Osloer Strasse, Brunnenstrasse North, and Wedding Center, have steadily increased overall, and in 2014 the proportion of children having any caries experience in Osloer Strasse reached 79%. This share of caries‐experienced children is extremely high compared to 20% at which Germany aims by 2020 (Ziller et al., 2006). Those three quarters have also shown constantly high numbers of untreated decayed teeth, while the averages of other quarters in Mitte have generally decreased. Among the former, the average of untreated decayed teeth in Osloer Strasse has risen consistently since 2008 and reached 2.04 in 2014. The oral health gap between the quarters, which has been gradually widening over time, can also be identified for the presence of any caries experience and untreated caries.

**FIGURE 5:**
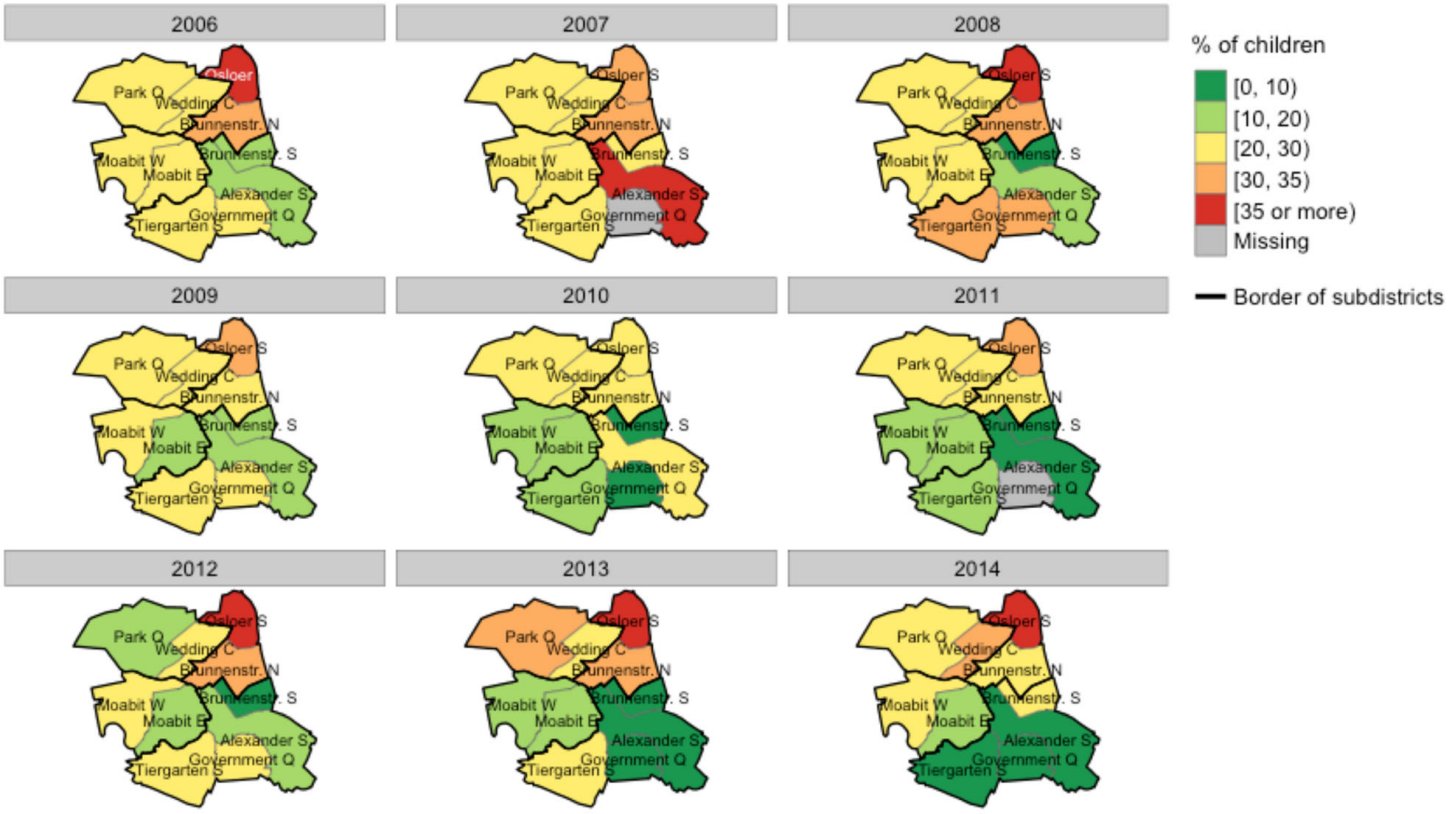
Spatio‐temporal variation of caries risk at quarter level in Berlin‐Mitte from 2006 to 2014. Black boundaries indicate the four subdistricts of Mitte; Gray boundaries indicate the ten quarters of Mitte

The distribution of children with high caries risk over time is mapped in Figure 5. Patterns similar to those in the previous two outcomes were also monitored in caries risk. Risk groups were primarily found in the quarters of Osloer Strasse, Brunnenstrasse North, and Wedding Center. Of particular interest is that the proportion of children with caries risk in Mitte increased by more than 4% in 2014 compared to 2013, though the number of quarters with a high proportion of risk groups decreased. Moreover, the percentage of risk groups in the northern part of Mitte centered at Osloer Strasse and Wedding Center has grown rapidly since 2010, whereas that in the southern part of Mitte has decreased. A horizontal zone of a generally good level of oral health mentioned before can also be seen on this map, which is crossing the center of Mitte. These areas have had very low rates of caries risk over the study period except for years with very few participants.

## 4 DISCUSSION

### 4.1 Summary of findings

This study conducted an exploratory analysis at the small‐area level using routinely collected data and found a relevant gap in children's dental caries between the quarters of Berlin‐Mitte. Along with the variation in spatial distribution of oral health inequalities, a change in scale of the inequalities was also discovered. The three quarters Osloer Strasse, Brunnenstrasse North, and Wedding Center bordering one another are the areas where the lowest oral health status in Mitte was observed throughout the whole study period. For all four outcomes, the total mean of Mitte has risen over time in spite of steady improvements achieved in the southern part of Mitte. The worsening average of Mitte, contrary to reducing caries experience in Berlin and Germany, may be explained by the fact that the northeast quarters of Mitte (Osloer Strasse, Brunnenstrasse North, Wedding Center) have shown a sharp deterioration in caries experience, especially in the number of untreated decayed teeth. This implies spatially and temporally mounting disparities in children's oral health of Mitte (Butler et al., 2009; Splieth et al., 2017).

### 4.2 Comparison with other studies and a possible explanation for findings

Our results are consistent with a previous study (Watt & Sheiham, 1999), which reports large district and regional discrepancies in caries experience of children, and are in line with recent research suggesting that the place of residence is one of the social determinants of health and can thus help predicting health levels to some extent (Johnson, Hines, Johnson 3rd, & Bayakly, 2014; Marmot, 2010). There have been a number of studies indicating that socio‐demographic factors highly correlate with dental caries among children. According to earlier findings, strong associations were established between socio‐economic inequalities (i.e., in terms of ethnicity, wealth, parental education, and employment) and caries experience in children (Kramer, Petzold, Hakeberg, & Ostberg, 2018). An increase was also observed in the association between deprivation levels and dmft in 5‐year‐olds over time (comparing 2013 with 2003) (Masood, Mnatzaganian, & Baker, 2019). A systematic review supports the idea of an association between the parental educational and occupational background, income and having any caries lesions or experience (Schwendicke et al., 2015). A significant association between children's oral health and a migration background has also been noted in an earlier study (Cvikl et al., 2014).

The three quarters (Osloer Strasse, Brunnenstrasse North, Wedding Center) are the very areas where the percentages of unemployment, welfare recipients, and child poverty have been noticeably high (Foundation SPI, 2017). Above all, the share of children with a migration background in these quarters was exceptionally higher than in other quarters. An overwhelmingly large proportion (55–64%) of children from the three quarters were of Turkish and Arab origins (Appendix Figure 4). Their oral health status was at a lower level compared with other origin groups (Appendix Figure 5). Additionally, regarding the whole district of Mitte, sudden exacerbations among children of Eastern and Western European origins appear to account for much of the decline in oral health since 2012 as well (Appendix Figure 5). Another interesting point is the “not answered” group for the question on the country of origin. The average dmft of this group was the second highest (3.58 [SD 3.58]), just behind children of Eastern European origin (4.12 [SD 3.60]), even if considering the small number (78 children) of the group. They are likely to have parents with migration background unable to communicate in German as shown in previous research (Brabete, Doray‐Demers, & Vissandjée, 2020). For this reason, they have not been precisely identified in research so far and seem to constitute a blind spot of oral health. This group deserves further investigation.

### 4.3 Strengths and limitations

Our study analyzes dental caries in children based on a large, representative sample for Berlin‐Mitte. The use of routinely collected data covering the whole population of first‐year schoolchildren in Mitte facilitated the spatio‐temporal analysis at a small‐area level. Focusing on small areas at quarter and community levels helps to transfer the conventional perspective in dental research to the new dimension of small areas. As our findings reveal, the caries pattern at a small‐area level can be completely different from that at national or regional levels. By observing a small area, the study provides more specific information on where disadvantaged children in terms of oral health are located. Furthermore, using routinely collected data from a repeated cross‐sectional survey, changing characteristics of the district Mitte over time could be explored. As data of all children who participated in the health examination were analyzed, the risk of selection bias was lower compared to data obtained by other sampling methods.

One limitation of the study is that the demographic variables were limited to sex, age, and country of origin. Other socio‐economic factors, such as parental education, income or employment status were not controlled for in this study. Another limitation is that the numbers of children by quarter originally invited to the health examination were not available due to German data privacy regulations, and consequently response rates by quarter could not be calculated. However, except for the subdistrict Zentrum containing quarters of a very low population density, the numbers of participants from the other quarters were steady overall during the study period. For that reason and the high sample size of our study, we did not consider this as biasing spatial distribution of dental caries.

### 4.4 Policy implication

Caries in primary dentition is an important indicator for predicting future caries in permanent dentition (Li & Wang, 2002). Interventions to combat health inequalities should already begin in early childhood, during which higher returns on investment are expected than in adolescence (Marmot, 2010). In this context, a more effective and efficient approach for narrowing the oral health gap in Mitte would be to establish tailored programs focusing on those areas where high‐risk children live. First, preventive programs could be implemented in kindergarten or school settings, which are recognized as the best places to perform group prophylaxis (WHO, 2003). Many studies have shown the effectiveness of fluoride in children's oral health (Divaris, Rozier, & King, 2012; Slade, Grider, Maas, & Sanders, 2018), and recent systematic reviews confirmed the pronounced caries‐preventive benefit of fluoride mouthrinses and fluoride varnishes in primary teeth (Marinho, Chong, Worthington, & Walsh, 2016; Marinho, Worthington, Walsh, & Clarkson, 2013). Therefore, programs focusing on the use of fluoride mouthrinses or fluoride varnishes are valuable for children's oral health. Secondly, education programs on reducing sugar intake, which is one of the most important determinants associated with children's dental caries, are recommended. Previous studies have shown that high sugar consumption among children, for example through sweet desserts, snacks, and tea, results in increased caries experience (Elamin, Garemo, & Gardner, 2018; Sayegh, Dini, Holt, & Bedi, 2002). Since the pattern of sugar consumption is mostly influenced by parents, education programs for oral health promotion should be provided for both children and parents (Schneider, Jerusalem, Mente, & De Bock, 2013). In this way, it can correct the sweet preference of children and contribute to the development of good habits for children's oral health. Based on our findings, these programs should especially concentrate on quarters vulnerable to oral health problems, thereby decreasing disparities between the quarters. A small‐area approach is, in this regard, of practical relevance as our results also suggest. Customized programs planned on the basis of small‐area analysis need to be encouraged further to tackle the deterioration of children's oral health in certain small areas as a part of regional and national preventive measures.

## 5 CONCLUSION

Using routinely collected data, this study demonstrates that the oral health status among first‐year schoolchildren in Berlin‐Mitte has been worsening since 2010, contrary to the improving trend in Berlin and Germany, and that there are relevant inequalities between the quarters of Berlin‐Mitte. The usage of a small‐area approach enabled us to effectively reveal the spatial distribution of children's caries at the local level, providing a fundamental basis for more elaborate programs at the small‐area level regarding oral health promotion. Particular quarters in Mitte consistently presented the poorest oral health status in all four outcomes. Children having high caries risk have increasingly concentrated in these areas over time. This implicates the spatiotemporally widening gap in children's oral health in Mitte and underlines the necessity for local preventive strategies. Future caries research should therefore make preferential use of small‐area analysis, which could contribute to reducing the inequalities in children's oral health.

## CONFLICT OF INTEREST

The authors declare that they have no conflict of interest with regard to the authorship or publication of this article. TK reports outside the submitted work to have received honoraria from Lilly, Newsenselab, Total and The BMJ.

## AUTHORS' CONTRIBUTIONS

AKL contributed to conception, design, data acquisition, analysis, and interpretation. AA contributed to data analysis and interpretation. TS contributed to conception, design, and data analysis. TK contributed to conception, design, and data interpretation. AKL drafted the manuscript, and all authors provided critical feedback, discussed the results and commented on the manuscript.

## Supporting information


**Appendix** **S1**: Supporting Information

## Data Availability

Research data are not shared due to German data privacy restrictions.
