# Supplementary material for: Spatio‐temporal trends in caries: A study on children in Berlin‐Mitte
Source: Clin Exp Dent Res. 2020 Nov 17;7(2):196–204. doi: 10.1002/cre2.354 (PMC8019772; doi:10.1002/cre2.354)
Supplement: Supplementary file 1 — Appendix S1: Supporting Information [file CRE2-7-196-s001.zip › CRE2_354_cre2.20200101-File007.docx]

**< Appendices >**

**
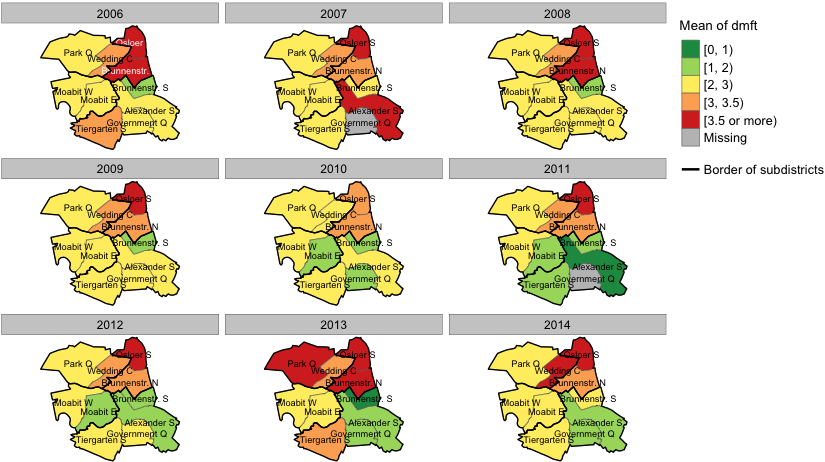
**

**Appendix Figure 1.** Spatio-temporal variation of mean dmft (decayed, missing, and filled teeth) at quarter level in Berlin-Mitte from 2006 to 2014. Black boundaries indicate the four subdistricts of Mitte; Gray boundaries indicate the ten quarters of Mitte.

**
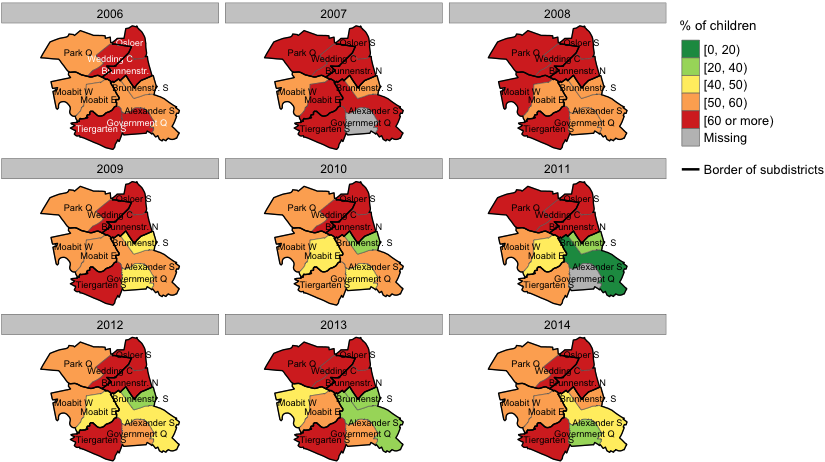
**

**Appendix Figure 2.** Spatio-temporal variation of the presence of any caries experience at quarter level in Berlin-Mitte from 2006 to 2014. Black boundaries indicate the four subdistricts of Mitte; Gray boundaries indicate the ten quarters of Mitte.

**
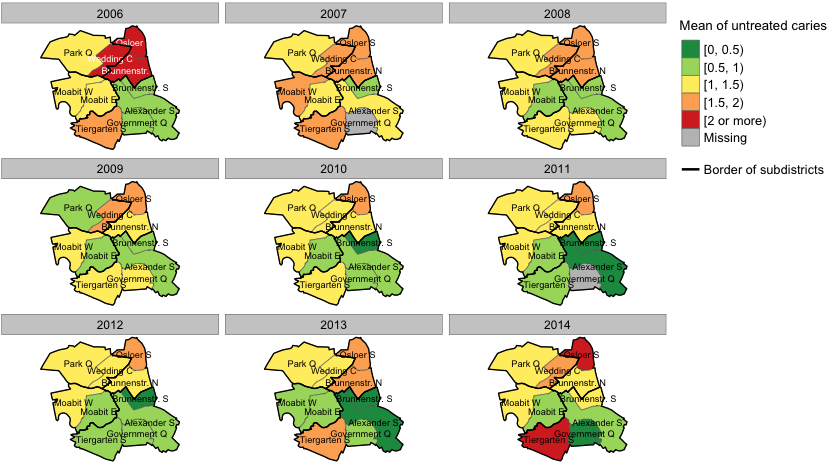
**

**Appendix Figure 3.** Spatio-temporal variation of untreated caries at quarter level in Berlin-Mitte from 2006 to 2014. Black boundaries indicate the four subdistricts of Mitte; Gray boundaries indicate the ten quarters of Mitte.


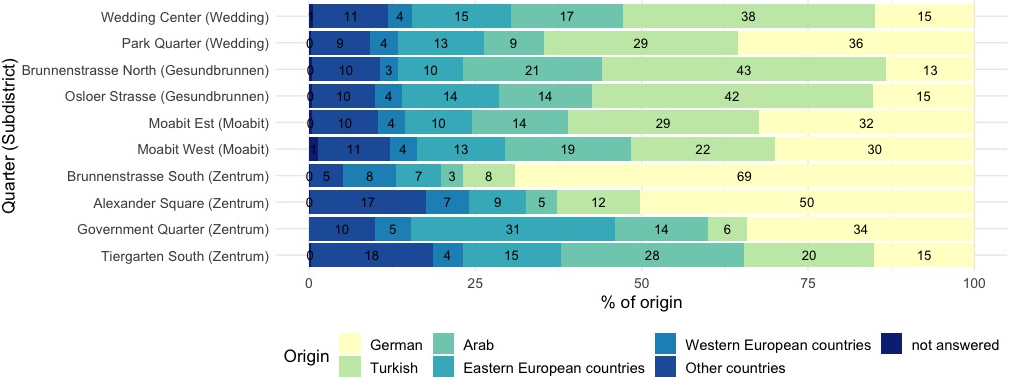


**Appendix Figure 4.** Proportion of children’s origin for each quarter in Berlin-Mitte during 2006-2014.


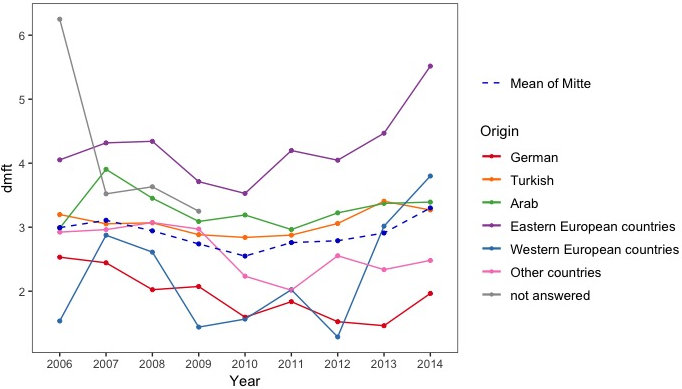


**Appendix Figure 5.** Trends in mean dmft (decayed, missing, and filled teeth) for each origin of children in Berlin-Mitte from 2006 to 2014.

**Appendix Table.** Characteristics of study population by year

|  | **Total** | **2006** | **2007** | **2008** | **2009** | **2010** | **2011** | **2012** | **2013** | **2014** |
| --- | --- | --- | --- | --- | --- | --- | --- | --- | --- | --- |
| **Total** | 14866 | 1719  (11.6) | 1144  (7.7) | 1714  (11.5) | 1927  (13.0) | 1989  (13.4) | 1587  (10.7) | 2016  (13.6) | 1658  (11.2) | 1112  (7.5) |
| **Sex (%)** |  |  |  |  |  |  |  |  |  |  |
| male | 7574 (50.9) | 878 (51.1) | 582 (50.9) | 869 (50.7) | 980 (50.9) | 993 (49.9) | 812 (51.2) | 1030 (51.1) | 851 (51.3) | 579 (52.1) |
| female | 7292 (49.1) | 841 (48.9) | 562 (49.1) | 845 (49.3) | 947 (49.1) | 996 (50.1) | 775 (48.8) | 986 (48.9) | 807 (48.7) | 533 (47.9) |
| **Age (%)** |  |  |  |  |  |  |  |  |  |  |
| 5 | 6424 (43.2) | 808 (47.0) | 508 (44.4) | 799 (46.6) | 829 (43.0) | 867 (43.6) | 662 (41.7) | 865 (42.9) | 720 (43.4) | 366 (32.9) |
| 6 | 8307 (55.9) | 899 (52.3) | 631 (55.2) | 904 (52.7) | 1081 (56.1) | 1092 (54.9) | 908 (57.2) | 1121 (55.6) | 933 (56.3) | 738 (66.4) |
| 7 | 135 (0.9) | 12 (0.7) | 5 (0.4) | 11 (0.6) | 17 (0.9) | 30 (1.5) | 17 (1.1) | 30 (1.5) | 5 (0.3) | 8 (0.7) |
| **Origin (%)** |  |  |  |  |  |  |  |  |  |  |
| German | 3802 (25.6) | 534 (31.1) | 270 (23.6) | 503 (29.4) | 568 (29.5) | 547 (27.5) | 349 (22.0) | 483 (24.0) | 375 (22.6) | 173 (15.6) |
| Turkish | 4720 (31.8) | 551 (32.1) | 471 (41.2) | 536 (31.3) | 609 (31.6) | 594 (29.9) | 518 (32.6) | 600 (29.8) | 473 (28.5) | 368 (33.1) |
| Arab | 2238 (15.1) | 134 (7.8) | 115 (10.0) | 201 (11.7) | 278 (14.4) | 298 (15.0) | 330 (20.8) | 339 (16.8) | 301 (18.2) | 242 (21.8) |
| Eastern European  countries | 1849 (12.4) | 192 (11.2) | 107 (9.4) | 187 (10.9) | 236 (12.2) | 288 (14.5) | 222 (14.0) | 343 (17.0) | 158 (9.5) | 116 (10.4) |
| Western European  countries | 616 (4.1) | 43 (2.5) | 24 (2.1) | 36 (2.1) | 66 (3.4) | 62 (3.1) | 42 (2.7) | 56 (2.8) | 182 (11.0) | 105 (9.4) |
| Other countries | 1563 (10.5) | 261 (15.2) | 134 (11.7) | 232 (13.5) | 138 (7.2) | 200 (10.1) | 126 (7.9) | 195 (9.7) | 169 (10.2) | 108 (9.7) |
| not answered | 78 (0.5) | 4 (0.2) | 23 (2.0) | 19 (1.1) | 32 (1.7) | 0 (0.0) | 0 (0.0) | 0 (0.0) | 0 (0.0) | 0 (0.0) |
| **Quarter (%)** |  |  |  |  |  |  |  |  |  |  |
| Tiergarten South | 312 (2.1) | 32 (1.9) | 44 (3.8) | 40 (2.3) | 40 (2.1) | 41 (2.1) | 34 (2.1) | 42 (2.1) | 38 (2.3) | 1 (0.1) |
| Government Quarter | 202 (1.4) | 26 (1.5) | 0 (0.0) | 19 (1.1) | 35 (1.8) | 42 (2.1) | 0 (0.0) | 37 (1.8) | 39 (2.4) | 4 (0.4) |
| Alexander Square | 809 (5.4) | 130 (7.6) | 2 (0.2) | 108 (6.3) | 183 (9.5) | 118 (5.9) | 2 (0.1) | 124 (6.2) | 129 (7.8) | 13 (1.2) |
| Brunnenstrasse South | 734 (4.9) | 91 (5.3) | 17 (1.5) | 201 (11.7) | 102 (5.3) | 110 (5.5) | 33 (2.1) | 115 (5.7) | 31 (1.9) | 34 (3.1) |
| Moabit West | 1764 (11.9) | 184 (10.7) | 165 (14.4) | 166 (9.7) | 221 (11.5) | 256 (12.9) | 250 (15.8) | 227 (11.3) | 191 (11.5) | 104 (9.4) |
| Moabit Est | 1683 (11.3) | 176 (10.2) | 152 (13.3) | 193 (11.3) | 201 (10.4) | 235 (11.8) | 237 (14.9) | 231 (11.5) | 184 (11.1) | 74 (6.7) |
| Osloer Strasse | 2247 (15.1) | 276 (16.1) | 211 (18.4) | 224 (13.1) | 242 (12.6) | 235 (11.8) | 245 (15.4) | 293 (14.5) | 268 (16.2) | 253 (22.8) |
| Brunnenstrasse North | 2468 (16.6) | 196 (11.4) | 209 (18.3) | 275 (16.0) | 314 (16.3) | 322 (16.2) | 278 (17.5) | 332 (16.5) | 292 (17.6) | 250 (22.5) |
| Park Quarter | 1616 (10.9) | 185 (10.8) | 83 (7.3) | 182 (10.6) | 208 (10.8) | 254 (12.8) | 164 (10.3) | 240 (11.9) | 176 (10.6) | 124 (11.2) |
| Wedding Center | 3031 (20.4) | 423 (24.6) | 261 (22.8) | 306 (17.9) | 381 (19.8) | 376 (18.9) | 344 (21.7) | 375 (18.6) | 310 (18.7) | 255 (22.9) |
